# Prediction of Kidney Function Improvement After Heart Transplantation

**DOI:** 10.3390/biomedicines13040933

**Published:** 2025-04-10

**Authors:** Jakub Ptak, Mateusz Sokolski, Mateusz Wilk, Mateusz Waloszczyk, Kacper Wiśniewski, Dominik Krupka, Paulina Makowska, Magdalena Cielecka, Maciej Szwajkowski, Mateusz Rakowski, Maciej Bochenek, Roman Przybylski, Michał Zakliczyński

**Affiliations:** 1 Institute of Heart Diseases, Jan Mikulicz Radecki University Hospital Wroclaw, 50556 Wroclaw, Poland; jakubptak.jp2@gmail.com (J.P.); magdacielecka@wp.pl (M.C.); mateusz.rakowski@usk.wroc.pl (M.R.);; 2 Clinical Department of Heart Transplantation and Mechanical Circulatory Support, Department of Cardiac, Surgery and Heart Transplantation, Institute of Heart Diseases, Faculty of Medicine, Wroclaw Medical University, 50368 Wroclaw, Poland; 3 Institute of Heart Diseases, Student Scientific Club of Transplantology and Advanced Therapies of Heart Failure, Faculty of Medicine, Wroclaw Medical University, 50368 Wroclaw, Poland; wilk.mateusz1@gmail.com (M.W.); mateusz114442@gmail.com (M.W.);; 4 Department of Gastroenterology and Internal Medicine, Medical University of Warsaw, 02097 Warszawa, Poland; 5 Faculty of Health Sciences, Wroclaw Medical University, 50368 Wroclaw, Poland; 6 Department of Cardiac Surgery and Heart Transplantation, Institute of Heart Diseases, Faculty of Medicine, Wroclaw Medical University, 50368 Wroclaw, Poland

**Keywords:** heart failure, heart transplantation, cardio-renal syndrome, glomerular filtration rate, worsening renal function, post-transplant outcomes

## Abstract

**Background/Objectives**: Patients with advanced heart failure (HF) often suffer from impaired kidney function. Based on the pathophysiology of types I and II of cardiorenal syndrome, heart transplantation (Htx) may restore renal function. The aim of this study was to identify predictors of improvement in kidney function after HTx. **Methods**: Htx patients from a tertiary hospital were retrospectively divided into three groups—improvement (n = 24), deterioration (n = 31) and no significant change in eGFR (n = 45)—based on changes in their mean estimated glomerular filtration rate (eGFR) within the first three postoperative months, compared to the last three preoperative months. The threshold for eGFR improvement was defined as a ≥20% increase, while deterioration was defined as a ≥20% decrease. The no significant change group was defined as any change falling between these two values. **Results**: The median age of analyzed cohort was 54 (45–63) years, and 82% were male. Preoperatively, the improvement group was more frequently treated with inotropes or vasopressors and had significantly higher blood urea and total bilirubin levels before Htx. In the multivariate analysis, total bilirubin before Htx (OR 1.66; 95% CI; 1.24–2.69; *p* = 0.002) and no need for RRT early after Htx (OR 0.46; 95% CI 0.24–0.88; *p* = 0.02) were independent predictors of improved kidney function in the first three months after HTx. **Conclusions**: The improvement in renal function after HTx is uncommon. It could be expected in patients suffering from more severe forms of HF, with impaired kidney and liver function but who did not need RRT after the surgery.

## 1. Introduction

Kidney insufficiency is a common comorbidity among patients suffering from heart failure (HF), occurring in up to 49% of this group [1]. It additionally worsens their condition, leading to further complications, hospital readmissions and greatly increased mortality [2,3]. The pathophysiological links between the heart and kidneys have been collectively encapsulated in the term cardiorenal syndrome (CRS). The Acute Dialysis Quality Initiative consensus group formalized a definition and proposed a classification of CRS in which it was divided into five clinical subtypes. In the context of our study types 1 and 2, characterizing kidney damage resulting from HF, are relevant. Based on their pathophysiology, with a key role for cardiac dysfunction, they could potentially be reversed after heart transplantation (HTx). To date, a lower pretransplant estimated glomerular filtration rate (eGFR), lack of hypertension, no preoperative use of mechanical circulatory support and a low preoperative cardiac index were associated with improved eGFR after the HTx [4,5,6].

However, instead of recovery, renal dysfunction is a common aftermath of HTx [7,8,9]. Preoperative kidney disease, acute kidney injury short after the operation, postoperative septic episodes, use of nephrotoxic antibiotics and calcineurin inhibitors along with presence of other diseases, including diabetes or hypertension, were named as possible explanations of this complication [10].

The factors influencing renal recovery after HTx remain incompletely understood. This makes it difficult to identify patients suffering from reversible forms of CRS, i.e., those who would experience additional benefits from a transplanted heart. A better understanding of this issue would help distinguish between patients who should also receive kidney transplants and those whose renal function is likely to improve after heart transplantation alone. Therefore, our aim was to investigate renal function changes in our center’s HTx patient population and to determine which patients showed improvement.

## 2. Materials and Methods

**Figure 1:**
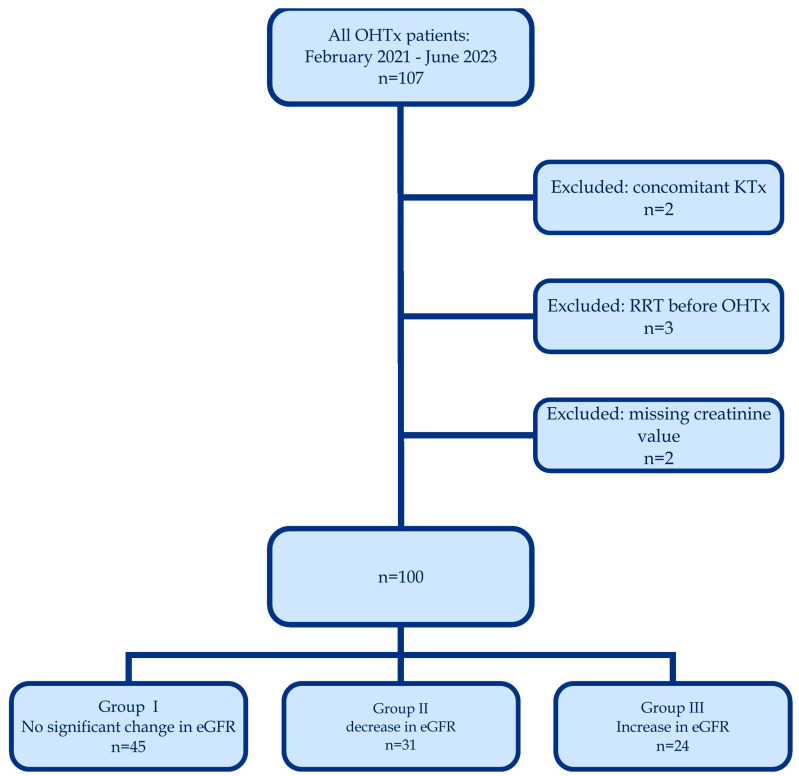
A flow chart illustrating the inclusion and exclusion criteria.

We retrospectively evaluated all patients who underwent HTx in a tertiary hospital between 25 February 2021 and 26 June 2023. Patients with concomitant kidney transplantation or on renal replacement therapy (RRT) before the HTx were excluded. Patients with missing preoperative or postoperative data were eliminated as well. A flow chart illustrating the inclusion and exclusion criteria is shown in Figure 1.

Collected preoperative data included demographics, comorbidities, chronic medication use, right heart catheterization measurements, 6 min walk test distance, cardiopulmonary exercise test results, the New York Heart Association (NYHA) and Interagency Registry for Mechanically Assisted Circulatory Support (INTERMACS) classes, donor characteristics and laboratory parameters. Postoperative data included surgical complications, the need for renal replacement therapy and laboratory parameters.

Baseline laboratory values were defined as the mean from the three months preceding HTx. Mean values from the three months after the operation were defined as postoperative.

We assessed renal function as eGFR calculated with the Modification of Diet in Renal Disease (MDRD) formula. MDRD formula was reported to be the most precise and accurate in the group of heart and other solid organ transplantation recipients [11,12]. Based on the difference between the baseline and postoperative eGFR, we distinguished three groups:

I—no significant change in renal function, defined as change within −20% and +20% range (n = 45).

II—significant worsening of renal function, defined as ≥20% decrease (n = 31).

III—significant improvement in renal function, defined as ≥20% increase (n = 24).

We compared the groups in terms of their preoperative and postoperative characteristics and identified predictors of significant improvement in renal function.

Statistical methods:

We performed a descriptive analysis with mean and standard deviation or median and interquartile range for continuous parametric and nonparametric data. Qualitative variables were presented as absolute frequencies with percentages. We compared continuous variables using ANOVA and Kruskal–Wallis ANOVA for parametric and nonparametric data. Qualitative variables were compared with χ^2^ test.

We utilized a univariate regression to assess potential predictors of improvement in kidney function. Then, after exclusion of variables with *p* > 0.05, a stepwise backward selection was used to fit a multivariate logistic regression model. The model was internally validated using v-fold cross-validation technique. The accuracy of the model was evaluated with the area under the receiver operating characteristic curve.

A *p* value below 0.05 was considered significant. We performed all the statistical analyses with Statistica 13.3 (Tibco, Palo Alto, CA, USA).

## 3. Results

### The Results Description

Of the total 107 identified patients, 100 were included in the final analysis. Two recipients of concomitant kidney transplant, three patients on RRT before HTx and two patients with missing creatinine values from preoperative or postoperative period were excluded. A total of 82% of the analyzed cohort were male, with median age of 54 (45–63) years. Sixteen patients (16%) died within the first three months after HTx. Median preoperative eGFR of the whole population was 68 (56–84) mL/min/1.73 m^2^. Patients’ clinical and demographic characteristics are presented in Table 1. Donors’ and perioperative data are presented in Table S1 in Supplementary Materials.

Forty-five (45%) patients had no significant change in eGFR (group I). Thirty-one (31%) suffered from a significant drop in eGFR (group II). Twenty-four patients (24%) experienced a significant increase in eGFR compared to baseline (group III). Preoperative and postoperative laboratory data are summarized in Table 2 and Table 3, respectively.

Preoperative inotropes or vasopressors use (*p* = 0.04), total bilirubin (*p* = 0.0003) and urea levels (*p* = 0.04) differed significantly between the three groups. Baseline total bilirubin in group III was significantly higher than in the other two groups, while baseline urea was significantly higher only compared to the group II. Differences in preoperative NYHA class, eGFR and need for postoperative RRT did not reach the statistical significance (*p* = 0.051, *p* = 0.052 and *p* = 0.079, respectively). In-hospital course data are shown in Table 4.

There were no significant differences in age, sex, BMI, smoking status, preoperative comorbidities, chronic medication use, right heart catheterization measurements, 6 min walk test distance, cardiopulmonary exercise test results, complications of the surgery or donor characteristics.

Within the first three months after the surgery, mean eGFR did not change in the whole cohort: 68 (56–84) vs. 66 (50–83) mL/min/1.73 m^2^ (*p* = 0.334). Similarly, there was no change in eGFR in group I: 66 (51–79) vs. 66 (52–75) mL/min/1.73 m^2^ (*p* = 0.394).

Group II experienced a 32% decline in eGFR: from 78 (62–96) to 53 (41–62) mL/min/1.73 m^2^ (*p* < 0.001). Group III, on the other hand, had a 33% increase in eGFR—from 66 (55–79) to 88 (78–114) mL/min/1.73 m^2^ (*p* < 0.001), along with a significant drop in total bilirubin from 2.2 (1.5–3) to 1.4 (0.96–2.1) mg/dL (*p* = 0.012).

In the univariate analysis preoperative urea, total bilirubin, NT-proBNP, ALP, ALT and no need for RRT in the immediate postoperative period were significantly associated with improving renal function. However, in the multivariate analysis, only total bilirubin (OR 1.66; 95% CI; 1.24–2.69; *p* = 0.002) and no need for RRT (OR 0.46; 95% CI 0.24–0.88; *p* = 0.02) were independent predictors of increase in kidney function. Model summary is presented in Table 5.

The most common infectious agents were Klebsiella pneumoniae (29%, NDM-19%, ESBL-10%), *Candida* spp. (14%) and Stenotrophomonas maltophilia (10%).

## 4. Discussion

Our study demonstrated that overall renal function, measured as eGFR, did not change significantly in the first three months after HTx, but we were able to distinguish the group of patients who showed renal function improvement. Higher baseline total bilirubin and no need for renal replacement therapy in the immediate postoperative period were independent predictors of renal improvement. Moreover, the increase in eGFR in this subgroup of patients was accompanied by a decrease in total bilirubin. Preoperatively, patients whose eGFR increased had significantly higher blood urea and total bilirubin levels and were more frequently treated with inotropes or vasopressors. Numerous studies focused on worsening renal function after HTx. However, ours is one of the few that concentrate on renal function recovery.

Patients whose renal function improved postoperatively had previously presented with a more advanced stage of heart failure, accompanied by worse baseline kidney function, higher total bilirubin levels and a more frequent need for inotropic or vasopressor support. It is consistent with previous research, showing that renal improvement occurs primarily in patients with poorer baseline eGFR [5,6,13], who are usually in worse overall condition [4,13]. However, to best of our knowledge, preoperative liver function has not yet been directly associated with improvement in eGFR. Our findings indicate potential mechanisms of worsening renal function that may allow for improvement. First, congestion and increased venous pressure with impaired transrenal gradient are potential causes of changes in intra-renal venous flow patterns [14,15]. Second, a pathophysiological pathway is a low output with low renal perfusion, which is linked with the use for inotropic support before HTx. The most often used inotrope in our study was milrionone, which has been associated with lower mortality and improved hemodynamics compared with dobutamine in patients with acute decompensated HF cardiogenic shock [16]. We found that higher baseline total bilirubin was an independent predictor of an increase in eGFR. Total bilirubin was shown to be associated with more severe HF [17] and to be a marker of venous congestion in the setting of chronic HF [18]. This suggests that improvement in renal function occurred in patients with multiorgan damage due to congestive HF. This is underlined by the fact that total bilirubin decreased significantly after HTx, alongside improvements in eGFR. However, we found no connection between central venous pressure or cardiac index and improved renal function, as previously reported [4]. It is possible that it is not only hemodynamic parameters, but also the presence of organ impairment caused by congestion helps identify patients who could experience renal function improvement.

No need for RRT in the immediate postoperative period was the second independent predictor of improving renal function. This finding aligns with research showing a more rapid decline in eGFR, with markedly worse renal function at 3 months post-transplant, in patients requiring RRT [19]. Similarly, the use of RRT was identified as a risk factor for developing end-stage renal disease [20]. Interestingly, poorer preoperative renal function is cited as a risk factor for the need to initiate RRT [19,21,22]. On the other hand, it favors an improvement in eGFR after HTx. This highlights the importance of distinguishing between patients suffering from CRS and those with other causes of renal impairment, as both groups would have lowered preoperative eGFR.

To the best our knowledge, only two studies focused on identifying predictors of renal function improvement following HTx to date. However, the one by Baudry et al. had hemodynamic parameters in the center of attention [4]. Compared to the research by Ivey-Miranda et al., which had a design similar to our study [6], we were able to gather a larger population and set a threshold for improved kidney function at a 20% change in eGFR instead of 10%. We believe that this cut-off value more accurately reflects a substantial change in the filtration rate. The differences in design may partially explain the discrepancies in our results. Nevertheless, it is also important to bear in mind that our study was single-center, with a relatively small sample size, which could influence the results. In addition, we did not analyze potential differences in postoperative drug usage between patients. The majority of patients were treated with a calcineurin inhibitor, mycophenolate mofetil and steroids, with preoperative induction by basiliximab. Furthermore, we used eGFR to assess renal function. It has been suggested that the agreement between eGFR and measured GFR (mGFR) was very low in HTx recipients [23]. Conversely, there were studies presenting the same trajectory of renal function following HTx, assessed by eGFR and mGFR [11,24].

We chose the first three months to evaluate postoperative kidney function. We believe it is a good starting point for assessing potential for renal improvement. It was shown that eGFR already stabilizes within this period [25,26] and its higher increase in the first postoperative month was associated with >15% improvement after one year from the HTx [27].

Some laboratory parameters may be influenced by the patient’s nutritional status and muscle mass, as malnutrition and sarcopenia are common in individuals with advanced HF. Unfortunately, our study did not collect data on key indicators of nutritional status or sarcopenia, such as serum albumin levels, body mass index or muscle mass assessments. Additionally, we lacked data on postoperative hemodynamic parameters that could provide insight into the degree of congestion during the postoperative period. Incorporating these parameters in future studies could offer a more comprehensive understanding of their impact on renal function recovery. Moreover, the dynamic changes in NT-proBNP levels observed before and after HTx, particularly their relationship with kidney function recovery, warrant further investigation. Understanding the determinants of these trends may yield critical insights into the complex interplay between cardiac and renal function following HTx and help guide the development of targeted therapeutic strategies.

Our study has several limitations. First, the retrospective, single-center design limits the generalizability of the findings. Second, the sample size in each group was relatively small, reflecting the early phase of our center’s heart transplantation program. Third, the NYHA classification was unavailable for 17% of the patients due to the urgent nature of many transplants. Fourth, our observation period was limited to three months; however, we aimed to capture early renal function changes post-HTx, when hemodynamic stabilization is most pronounced. Additionally, detailed data on intraoperative factors (e.g., cardiopulmonary bypass time, inotrope duration), bleeding, blood product use and patient frailty were not systematically collected and were therefore unavailable for analysis. Microbiological data on infectious agents were only partially recorded. Finally, eGFR estimation rather than measured GFR was used to assess kidney function, which may affect precision.

## 5. Conclusions

We showed that improved renal function after HTx is not common. It could be expected in patients suffering from more severe forms of HF, with impaired kidney and liver function, but who do not require RRT after surgery. It seems clear that one needs to have a decreased preoperative eGFR to expect improvement. However, it remains a challenge to differentiate between patients suffering from CRS and those with other causes of kidney impairment which would not resolve after HTx. We hope that, despite its limitations, our study will contribute to a better understanding of the discussed issue and facilitate clinical decision-making.

## Figures and Tables

**Table 1:** Characteristics of patients.

	All pts (100)	Group 1 (45)	Group 2 (31)	Group 3 (24)	*p*
Demographic data:					
Sex—number of women N (%)	18 (18%)	8 (18%)	6 (19%)	4 (17%)	0.966
Age (years)	54 (45–63)	54 (47–62)	51 (44–64)	56.5 (46–64)	0.610
BMI (kg/m^2^)	26.07 ± 4.25	26.75 ± 4.15	25.75 ± 4.25	24.97 ± 4.7	0.413
Preoperative clinical data:					
Etiology of HF—number of ischemic HF N (%)	43 (43%)	22 (49%)	11 (36%)	10 (42%)	0.509
Use of inotropes/vasopressor N (%)	40 (40%)	23 (51%)	7 (23%)	10 (42%)	0.044
NYHA functional class:					0.051
1 and 2 N (%)	14 (14%)	4 (9%)	8 (26%)	2 (8%)	
3 N (%)	39 (39%)	22 (49%)	8 (26%)	9 (38%)	
4 N (%)	30 (30%)	11 (24%)	8 (26%)	11 (46%)	
INTERMACS profile					0.219
1–4 N (%)	36 (53%)	7 (10%)	18 (26%)	11 (16%)	
5–7 N (%)	32 (47%)	11 (16%)	16 (24%)	5 (7%)	
IABP/ECMO/LVAD/Impella N (%)	25 (25%)	12 (27%)	9 (29%)	4 (17%)	0.542
Smoking. either now or in the past N (%)	45 (45%)	22 (49%)	13 (42%)	10 (42%)	0.719
ICD/CRT-D N (%)	61 (61%)	29 (64%)	18 (58%)	14 (58%)	0.815
CVP (mmHg)	9 (6–14)	7 (4.5–15)	9 (6.5–12)	10 (9–17)	0.421
PAS (mmHg)	43.21 ± 14.38	42 ± 15.81	40.71 ± 14.97	47.69 ± 10.92	0.333
PAD (mmHg)	21 (16–26)	19.5 (15.5–24)	19 (16–30)	24 (19.5–27)	0.198
PAM (mmHg)	30 (23.5–36)	28 (21–33)	28 (21–37)	33 (29–37)	0.340
PCWP (mmH)g	20 (16–25)	20 (15–25)	19.5 (13.5–28)	23 (19–25)	0.424
CO (thermodilution) (L/min)	4.1 (3.5–4.7)	4.09 (3.5–4.7)	3.92 (3.47–4.4)	4.45 (3.56–5.3)	0.611
CI (thermodilution). L/min/m^2^	2.07 (1.78–2.44)	2.09 (1.84–2.44)	2.08 (1.75–2.27)	2.06 (1.78–2.70)	0.831
PAPI	2.2 (1.5–3.3)	2.4 (1.1–3.0)	2.8 (1.7–3.3)	2.9 (1.7–3.7)	0.119
RAP/PCWP	0.46 (0.33–0.66)	0.58 (0.35–0.80)	0.49 (0.31–0.65)	0.47 (0.40–0.65)	0.475
Comorbidities:					
Hypertension N (%)	45 (45%)	24 (53%)	13 (42%)	8 (33%)	0.259
Hyperlipidemia N (%)	41 (41%)	19 (42%)	11 (36%)	11 (46%)	0.723
Diabetes N (%)	27 (27%)	13 (29%)	7 (23%)	7 (29%)	0.800
Chronic kidney disease N (%)	27 (27%)	10 (22%)	12 (39%)	5 (21%)	0.208
Undergone CABG N (%)	13 (13%)	5 (11%)	6 (19%)	2 (8%)	0.425
Undergone PCI N (%)	42 (42%)	20 (44%)	14 (45%)	8 (33%)	0.613
Previous TIA/stroke N (%)	12 (12%)	6 (13%)	2 (7%)	4 (17%)	
AF/Afl N (%)	48 (48%)	19 (42%)	18 (58%)	11 (46%)	0.568

BMI—body mass index, HF—heart failure, NYHA—New York Heart Association, IABP—intra-aortic balloon pump, ECMO—extracorporeal membrane oxygenation, LVAD—left ventricular assist device, ICD—implantable cardioverter-defibrillator, CRT-D—cardiac resynchronization therapy—defibrillator, CVP—central venous pressure, PAS—pulmonary artery systolic pressure, PAD—pulmonary artery diastolic pressure, PAM—pulmonary artery mean pressure, pressure, PCWP—pulmonary capillary wedge pressure, CO—cardiac output, CI—cardiac index, CABG—coronary artery bypass grafting, PCI—percutaneous coronary interventions, TIA—transient ischemic attack, AF—atrial fibrillation, Afl—atrial flutter, RAP—right atrium pressure, PAPI—pulmonary pulsatility index.

**Table 2:** Preoperative laboratory data.

Preoperative Laboratory Data:	All Patients	Group 1	Group 2	Group 3	*p*
Hemoglobin (mg/dL)	12.72 ± 2.03	12.54 ± 2.04	12.84 ± 1.91	13.1 ± 2.05	0.522
Platelets (1000/mL)	194 (161–240)	200 (165–235)	194 (150–269)	179 (147–227)	0.505
INR	1.3 (1.2–1.7)	1.3 (1.1–1.5)	1.4 (1.1–1.8)	1.4 (1.2–1.8)	0.686
APTT (s)	38 (33–46)	37 (34–45)	36 (31–54)	39 (30–52)	0.820
Proteinuria (g)	0.167 (0.1–0.875)	0.1 (0.1–0.76)	0.3 (0.1–1.09)	0.3 (0.1–0.74)	0.192
Total bilirubin (mg/dL)	1.27 (0.8–1.98)	1.05 (0.78–1.92)	0.95 (0.74–1.54)	2.23 (1.51–2.99)	0.000
ALT (IU/L)	29.83 (20.64–62)	28.13 (20.13–38.47)	34.33 (23–46.57)	26.6 (19.55–349.97)	0.557
AST IU/L	39.67 (26.33–61.67)	37.8 (26.33–56)	41 (27.45–61.67)	36.86 (24.96–300.62)	0.730
ALP (IU/L)	85.71 (63.7–107.3)	82 (61.67–107.3)	87.5 (64–99)	91.33 (67.88–131)	0.423
GGTP (IU/L)	83.9375 (48.13–156.58)	84.5 (50.88–140)	69.25 (35.3–141.37)	115.83 (49–245)	0.442
Urea (mg/dL)	50 (38.85–66.21)	50.86 (40.8–66.25)	42 (33.81–58.64)	55.47 (44.67–75.54)	0.040
Creatinine (mg/dL)	1.13 (0.97–1.39)	1.18 (1–1.5)	1.05 (0.94–1.27)	1.25 (1–1.43)	0.128
eGFR (mL/min/1.73 m^2^)	68 (55.89–83.8)	65.72 (50.67–79)	77.57 (62.06–96.41)	66.28 (54.56–78.58)	0.052
Uric acid (mg/dL)	7.12 ±2.11	7.14 ± 2.27	7.12 ± 2.21	7.09 ± 1.73	0.996
Total protein (g/dL)	6.825 (6.12–7.3625)	7.03 (6.12–7.36)	6.95 (6–7.6)	6.7 (6.2–7.13)	0.754
Albumin (g/dL)	3.7 (3.07–4.1)	3.83 (3.33–4.14)	3.48 (2.95–4.15)	3.64 (3.11–3.87)	0.344
CRP (mg/L)	11.97 (3.56–50.95)	13.32 (5.06–46.85)	11.55 (2.35–54.92)	12.8 (3.05–55.77)	0.735
Total cholesterol (mg/dL)	126.86 ± 46.52	125.58 ± 31.11	130.4 ± 56.53	124.83 ± 58.61	0.921
LDL (mg/dL)	69.25 (48.25–92)	64 (48.5–74)	78 (66–116)	69.25 (45–103)	0.217
Natrium (mmol/L)	138 (137–140)	139 (137–140)	138 (136–140)	137 (136–139)	0.213
Potassium (mmol/L)	4.305 (4.091–4.5)	4.3 (4.13–4.5)	4.36 (4.07–4.58)	4.28 (4.06–4.51)	0.931
Total calcium (mg/dL)	9.04 (8.46–9.58)	9 (8.6–9.4)	9.4 (8.15–9.7)	9.4 (8.7–10.6)	0.232
Inorganic phosphate (mg/dL)	4.34 (3.2–5.1)	3.5 (3–5)	4.28 (3.48–5.8)	4.8 (3.2–5.1)	0.476
Procalcitonin (ng/L)	0.115 (0.043–0.45)	0.1 (0.04–0.29)	0.11 (0.05–0.54)	0.15 (0.03–0.7)	0.910
Troponin (ng/mL)	67 (16–1696)	26 (11–819)	246(26–2650)	113 (18–1553)	0.154
NTproBNP (pg/mL)	4219 (2228–8469)	3820 (2419–6331)	4244 (1587–6428)	5245 (2789–12701)	0.264
MELD 3.0 score	12.3 (8.6–17.2)	11.0 (7.3–12.6)	13.0 (7.5–17.1)	16.1 (8.9–20.1)	0.075

INR—international normalized ratio, APTT—activated partial thromboplastin clotting time, ALT—alanine aminotransferase, AST—aspartate aminotransferase, ALP—alkaline phosphatase, GGTP—gamma glutamyl transpeptidase, eGFR—estimated glomerular filtration rate, CRP—C-reactive protein, LDL—low-density lipoprotein, NTproBNP—N-terminal pro B-type natriuretic peptide.

**Table 3:** Postoperative laboratory data.

Postoperative Laboratory Data:	All Patients	Group 1	Group 2	Group 3	*p*
Hemoglobin (mg/dL)	10.75 ± 0.89	10.83 ± 0.94	10.62 ± 0.67	10.78 ± 1.07	0.482
Platelets (1000/mL)	209 (158–259)	210 (156–264)	209 (160–293)	201 (154–245)	0.474
INR	1.14 (1.09–1.23)	1.15 (1.09–1.22)	1.13 (1.08–1.3)	1.14 (1.1–1.2)	0.994
APTT (s)	32 (30–36)	32 (31–36)	33 (29–37)	32 (30–36)	0.764
Proteinuria (g)	0.17 (0.1–0.3)	0.17 (0.1–0.3)	0.19 (0.1–0.28)	0.1 (0.1–0.47)	0.806
Total bilirubin (mg/dL)	1.06 (0.68–1.58)	0.99 (0.63–1.51)	0.91 (0.65–1.18)	1.41 (0.96–2.08)	0.021
ALT (IU/L)	38.15 (26.07–69.75)	38.07 (26.08–58.46)	33.63 (21.15–105.71)	40.36 (32.21–78.46)	0.781
AST (IU/L)	38.23 (30.6–57.61)	37.27 (30–57.61)	38.71 (31.25–51.89)	38.44 (31.37–61.14)	0.813
ALP (IU/L)	95.71 (79.57–125.17)	91.22 (80.69–123.86)	96.46 (73.67–106.05)	104.73 (79.71–170.5)	0.356
GGTP (IU/L)	98.38 (67.14–156.6)	112.28 (72–156.6)	76.79 (61.63–122.71)	133.92 (83.32–265)	0.042
Urea (mg/dL)	66.35 (50–86)	68.54 (50–85.25)	69.5 (57.93–90.47)	58.02 (41.86–75.17)	0.102
Creatinine (mg/dL)	1.33 (1.07–1.74)	1.31 (1.1–1.68)	1.67 (1.36–2.22)	0.95 (0.76–1.21)	0.000
eGFR (ml/min/1.73 m^2^)	66 (49.73–83.26)	66 (52.37–74.93)	52.62 (40.5–61.82)	88.37 (77.97–114.36)	0.000
Uric acid (mg/dL)	5.96 ±1.56	6.1 ± 1.75	6.32 ± 1.33	5.18 ± 1.28	0.034
Total protein (g/dL)	5.97 (5.5–6.39)	6.13 (5.82–6.4)	5.88 (5.47–6.33)	5.6 (4.86–6.43)	0.020
Albumin (g/dL)	3.32 (3.09–3.58)	3.35 (3.13–3.64)	3.35 (2.88–3.57)	3.26 (2.95–3.59)	0.646
CRP (mg/L)	39.9 (26.01–65.39)	44.55 (30.04–65.79)	43.28 (26.91–81.71)	30.11 (20.79–48.64)	0.127
Total cholesterol (mg/dL)	167.07 ± 40.74	161.82 ± 38.59	171.32 ± 46.43	171.78 ± 39.91	0.578
LDL (mg/dL)	95 (75–121.5)	88.5 (74–109.25)	106 (71.25–126)	100.75 (89.08–136)	0.254
Natrium (mmol/L)	138 (137–139)	138(137–139)	138 (136–140)	138 (136–140)	0.881
Potassium (mmol/L)	4.46 (4.23–4.68)	4.42 (4.24–4.57)	4.49 (4.24–4.72)	4.49 (4.17–4.66)	0.681
Total calcium (mg/dL)	8.86 (8.45–9.17)	9.01 (8.56–9.27)	8.74 (8.3–9.02)	8.78 (8.49–9.1)	0.187
Inorganic phosphate (mg/dL)	3.99 (3.53–4.83)	3.85 (3.5–4.55)	4.83 (4.07–5.4)	3.37 (2.94–4.29)	0.000
Procalcitonin (ng/L)	1.35 (0.4–3.22)	1.17 (0.45–2.86)	2.2 (0.43–5.18)	0.75 (0.32–2.05)	0.108
Troponin (ng/mL)	4325(2896–7267)	4533.5 (2989–8725)	4584 (3158–7267)	3711(1399–6428)	0.328
NTproBNP (pg/mL)	5837 (3086–9044)	5979 (3624–9044)	7068 (3944–14168)	3820 (1920–8322)	0.155
MELD 3.0 score	6.9 (4.1–9.5)	7.6 (5.7–11.0)	6.3 (3.7–10.0)	5.4 (2.6–7.4)	0.066
Tacrolimus level (ng/mL)	11.3 (9.4–13.9)	11.7 (9.8–14.1)	11.8 (9.0–14.1)	11.2 (10.1–13.0)	0.974
Mycophenolate mofetil (ug/mL)	1.90 (1.1–2.5)	2.1 (1.6–2.5)	1.6 (1.2–2.4)	2.3 (0.9–2.8)	0.670

INR—international normalized ratio, APTT—activated partial thromboplastin clotting time, ALT—alanine aminotransferase, AST—aspartate aminotransferase, ALP—alkaline phosphatase, GGTP—gamma glutamyl transpeptidase, eGFR—estimated glomerular filtration rate, CRP—C-reactive protein, LDL—low-density lipoprotein, NTproBNP—N-terminal pro B-type natriuretic peptide

**Table 4:** In-hospital course.

In-Hospital Course Data:	All Patients	Group 1	Group 2	Group 3	*p*
Days in hospital	36 (25–60)	35 (25–55)	34 (22–57)	47 (31–78)	0.1623
Days in ICU	7 (5–13)	7 (4–14)	8 (6–13)	7 (4.5–10.5)	0.5085
Elective vs emergency (number of elective HTx)	17 (17%)	8 (18%)	7 (23%)	2 (8%)	0.3479
RRT after 3 months	4 (4%)	1 (2%)	2 (7%)	1 (4%)	
Deaths within 3 months (number of patients)	16 (16)	6 (13%)	5 (16%)	5 (21%)	0.7439
Postoperative RRT (number of patients)	40 (40%)	21 (47%)	14 (45%)	5 (21%)	0.0785
Infectious complications (number of patients)	21 (21%)	11 (24%)	4 (13%)	6 (25%)	0.1273
Rethoracotomy (number of patients)	23 (23%)	10 (22%)	8 (26%)	5 (21%)	0.9626
Right ventricular failure (number of patients)	2 (2%)	0 (0%)	1 (3%)	1 (4%)	0.4213
Pacemaker implantation (number of patients)	6 (6%)	3 (5%)	1 (3%)	2 (8%)	0.7615

ICU—intensive care unit, HTx—heart transplantation, RRT—renal replacement therapy.

**Table 5:** Univariate and multivariate predictors of renal improvement.

	Number of Patients	Univariate		Multivariate	
Variable		OR (95% CI)	*p*	OR (95% CI)	*p*
Sex—number of women (number of patients)	100	0.94 (0.51–1.73)	0.85		
Age (years)	100	0.89 (0.86–1.05)	0.76		
BMI (kg/m^2^)	100	0.92 (0.79–1.07)	0.3010		
Etiology of HF—number of ischemic HF (number of patients)	100	0.94 (0.59–1.5)	0.80		
Use of inotropes/vasopressor number of patients)	100	1.05 (0.66–1.67)	0.85		
NYHA functional class: (83)	83				
1 and 2 (number of patients)		0.98 (0.46–2.06)	0.95		
3 (number of patients)		1.89 (0.19–1.56)	0.10		
4 (number of patients)		0.54 (0.19–1.56)	0.26		
IABP/ECMO/LVAD/Impella (number of patients)	100	0.7 (0.39–1.27)	0.24		
CVP (mmHg)	67	1.05 (0.96–1.16)	0.3030		
PAS (mmHg)	67	1.03 (0.99–1.08)	0.1460		
PAD (mmHg)	67	1.03 (0.96–1.1)	0.3910		
PAM (mmHg)	67	1.03 (0.97–1.09)	0.3090		
PCWP (mmH)g	67	1.07 (0.98–1.17)	0.1530		
CO (thermodilution), (l/min)	67	1.04 (0.93–1.17)	0.4830		
CI (thermodilution), l/min/m^2^	67	0.88 (0.27–2.82)	0.8300		
Hypertension (number of patients)	100	0.73 (0.45–1.17)	0.19		
Hyperlipidemia (number of patients)	100	1.14 (0.72–1.81)	0.58		
Diabetes (number of patients)	100	1.07 (0.65–1.79)	0.78		
Chronic kidney disease (number of patients)	100	0.8 (0.46–1.39)	0.44		
Undergone CABG (number of patients)	100	0.73 (0.33–1.62)	0.44		
Undergone PCI (number of patients)	100	764 (0.49–1.27)	0.33		
Hemoglobin (mg/dL)	100	1.12 (0.89–1.41)	0.3450		
Platelets (1000/mL)	100	1 (0.99–1)	0.2550		
INR	100	1.48 (0.66–3.31)	0.3430		
APTT (s)	100	1 (0.97–1.03)	0.8830		
Proteinuria (g)	100	0.73 (0.24–2.18)	0.5710		
Total bilirubin (mg/dL)	100	1.66 (1.15–2.38)	0.0070	1.66 (1.24–2.69)	0.002
ALT (IU/L)	100	1 (1-1)	0.0480		
AST, (IU/L)	100	1 (1-1)	0.0690		
ALP (IU/L)	100	1.01 (1–1.02)	0.0270		
GGTP (IU/L)	100	1 (1–1.01)	0.1130		
Urea (mg/dL)	100	1.02 (1–1.04)	0.0510		
Creatinine (mg/dL)	100	1.99 (0.71–5.56)	0.1920		
eGFR (mL/min/1.73 m^2^)	100	0.99 (0.97–1.01)	0.2050		
Uric acid (mg/dL)	100	0.99 (0.78–1.25)	0.9380		
Total protein (g/dL)	100	0.94 (0.54–1.65)	0.8290		
Albumin (g/dL)	100	0.81 (0.37–1.76)	0.5890		
CRP (mg/L)	100	1 (0.98–1.01)	0.6940		
Total cholesterol (mg/dL)	100	1 (0.99–1.01)	0.8400		
LDL (mg/dL)	100	1 (0.99–1.02)	0.6940		
Natrium (mmol/L)	100	0.91 (0.79–1.05)	0.2040		
Potassium (mmol/L)	100	1.01 (0.98–1.04)	0.5300		
Total calcium (mg/dL)	100	2.12 (0.95–4.74)	0.0680		
Inorganic phosphate (mg/dL)	100	1.13 (0.67–1.92)	0.6440		
Procalcitonin (ng/L)	100	1.15 (0.96–1.39)	0.1280		
Troponin (ng/mL)	100	1 (1-1)	0.6310		
NTproBNP (pg/mL)	100	1 (1-1)	0.0330		
Days in hospital	100				
Days in ICU	100	1.03 (1–1.06)	0.0640		
Elective vs emergency (number of elective HTx)	100				
Postoperative RRT (number of patients)	100	0.55 (0.32–0.94)	0.03	0.46 (0.24–0.88)	0.02
Infectious complications (number of patients)	100	1.16 (0.68–2)	0.58		
Rethoracotomy (number of patients)	100	1 (0.56–1.77)	1.00		

BMI—body mass index, HF—heart failure, NYHA—New York Heart Association, IABP—intra-aortic balloon pump, ECMO—extracorporeal membrane oxygenation, LVAD—left ventricular assist device, CVP—central venous pressure, PAS—pulmonary artery systolic pressure, PAD—pulmonary artery diastolic pressure, PAM—pulmonary artery mean pressure, pressure, PCWP—pulmonary capillary wedge pressure, CO—cardiac output, CI—cardiac index, CABG—coronary artery bypass grafting, PCI—percutaneous coronary interventions, INR—international normalized ratio, APTT—activated partial thromboplastin clotting time, ALT—alanine aminotransferase, AST—aspartate aminotransferase, ALP—alkaline phosphatase, GGTP—gamma glutamyl transpeptidase, eGFR—estimated glomerular filtration rate, CRP—C-reactive protein, LDL—low-density lipoprotein, NTproBNP—N-terminal pro B-type natriuretic peptide, ICU—intensive care unit, HTx—heart transplantation, RRT—renal replacement therapy.

## Data Availability

All of the obtained data are presented in the tables.

## Supplementary Materials

The following supporting information can be downloaded at: https://www.mdpi.com/article/10.3390/biomedicines13040933/s1; Table S1: Donors and perioperative data.

## Abbreviations

The following abbreviations are used in this manuscript:

HF	Heart failure
CRS	Cardiorenal syndrome
HTx	Heart transplantation
eGFR	Estimated glomerular filtration rate
RRT	Renal replacement therapy
NYHA	New York Heart Association
INTERMACS	Interagency Registry for Mechanically Assisted Circulatory Support
MDRD	Modification of Diet in Renal Disease
ANOVA	Analysis of variance
BMI	Body mass index
NT-proBNP	N-terminal pro B-type natriuretic peptide
ALP	Alkaline phosphatase
ALT	Alanine aminotransferase
OR	Odds ratio
CI	Confidence interval
IABP	Intra-aortic balloon pump
ECMO	Extracorporeal membrane oxygenation
LVAD	Left ventricular assist device
ICD	Implantable cardioverter-defibrillator
CRT-D	Cardiac resynchronization therapy-defibrillator
CVP	Central venous pressure
PAS	Pulmonary artery systolic pressure
PAD	Pulmonary artery diastolic pressure
PAM	Pulmonary artery mean pressure
PCWP	Pulmonary capillary wedge pressure
CO	Cardiac output
CI	Cardiac index
CABG	Coronary artery bypass grafting
PCI	Percutaneous coronary interventions
TIA	Transient ischemic attack
AF	Atrial fibrillation
AFl	Atrial flutter
RAP	Right atrium pressure
PAPI	Pulmonary pulsatility index
INR	International normalized ratio
APTT	Activated partial thromboplastin clotting time
AST	Aspartate aminotransferase
GGTP	Gamma glutamyl transpeptidase
CRP	C-reactive protein
LDL	Low-density lipoprotein
ICU	Intensive care unit
mGFR	Measured glomerular filtration rate
