# Supplementary material for: Prediction of Kidney Function Improvement After Heart Transplantation
Source: Biomedicines. 2025 Apr 10;13(4):933. doi: 10.3390/biomedicines13040933 (PMC12025116; doi:10.3390/biomedicines13040933)
Supplement: Supplementary file 1 [file biomedicines-13-00933-s001.zip › biomedicines-3543045-supplementary.pdf]

	All pts (100)	Group 1 (45)	Group 2 (31)	Group 3 (24)	p
**Donors and perioperative data:**					
Sex - number of women N (%)	26 (26%)	12 (28%)	7 (24%)	7 (26%)	0.94
Age (years)	41 [34–49]	41 [35–48]	43 [33–50]	40 [34–45]	0.35
BMI (kg/m^2)	26.94 ± 3.65	25.72 ± 3.85	26.93 ± 3.44	24.85 ± 3.03	0.08
Cold ischemic time (minutes)	136 (107–170)	143 (119–167)	131 (113–156)	131 (113–156)	0.70
Aortic Cross-Clamp Time (minutes)	136 (81–180)	153 (76–183)	134 (80–171)	140 (83–188)	0.64
Perfusion Time (minutes)	177 (130–210)	170 (138–198)	168 (132–231)	168 (116–210)	0.83

- Aortic Cross-Clamp Time—duration during which the recipient's aorta is clamped during the transplantation procedure.
- Perfusion Time—total duration of cardiopulmonary bypass support in the recipient
- Cold Ischemic Time—the period from the application of the aortic cross-clamp in the donor until the donor heart is reperfused in the recipient
